# Intraductal cisplatin treatment in a *BRCA*-associated breast cancer mouse model attenuates tumor development but leads to systemic tumors in aged female mice

**DOI:** 10.18632/oncotarget.18490

**Published:** 2017-06-15

**Authors:** Jolien S. de Groot, Paul J. van Diest, Miranda van Amersfoort, Eva J. Vlug, Xiaojuan Pan, Natalie D. ter Hoeve, Hilde Rosing, Jos H. Beijnen, Sameh A. Youssef, Alain de Bruin, Jos Jonkers, Elsken van der Wall, Patrick W.B. Derksen

**Affiliations:** ^1^ Department of Pathology, University Medical Center Utrecht, Utrecht, The Netherlands; ^2^ Department of Pharmacy and Pharmacology, Netherlands Cancer Institute, Amsterdam, The Netherlands; ^3^ Department of Pharmaceutical Sciences, Utrecht University, Utrecht, The Netherlands; ^4^ Department of Pathobiology, Dutch Molecular Pathology Center, Faculty of Veterinary Medicine, Utrecht University, Utrecht, The Netherlands; ^5^ Department of Pediatrics, Division of Molecular Genetics, University Medical Center Groningen, University of Groningen, Groningen, The Netherlands; ^6^ Department of Molecular Pathology, Netherlands Cancer Institute, Amsterdam, The Netherlands; ^7^ Department of Medical Oncology, University Medical Center Utrecht, Utrecht, The Netherlands

**Keywords:** breast cancer, BRCA, intraductal, cisplatin, olaparib

## Abstract

*BRCA* deficiency predisposes to the development of invasive breast cancer. In *BRCA* mutation carriers this risk can increase up to 80%. Currently, bilateral prophylactic mastectomy and prophylactic bilateral salpingo-oophorectomy are the only preventive, albeit radical invasive strategies to prevent breast cancer in BRCA mutation carriers. An alternative non-invasive way to prevent *BRCA1*-associated breast cancer may be local prophylactic treatment via the nipple.

Using a non-invasive intraductal (ID) preclinical intervention strategy, we explored the use of combined cisplatin and poly (ADP)-ribose polymerase 1 (PARP1) inhibition to prevent the development of hereditary breast cancer. We show that ID cisplatin and PARP-inhibition can successfully ablate mammary epithelial cells, and this approach attenuated tumor onset in a mouse model of Brca1-associated breast cancer from 153 to 239 days. Long-term carcinogenicity studies in 150 syngeneic wild-type mice demonstrated that tumor incidence was increased in the ID treated mammary glands by 6.3% due to systemic exposure to cisplatin. Although this was only evident in aged mice (median age = 649 days), we conclude that ID cisplatin treatment only presents a safe and feasible local prevention option if systemic exposure to the chemotherapy used can be avoided.

## INTRODUCTION

Breast cancer risk increases dramatically in women carrying mutations in a breast cancer susceptibility gene, most frequently *BRCA1* or *BRCA2* [1]. Depending on the type of mutation and the presence of genetic modifiers, this risk can increase to more than 80% [2, 3].

Mammary gland architecture is dynamic and changes during puberty, pregnancy, lactation and involution post partum and post menopause [4, 5]. At puberty, the mouse mammary ductal system starts growing from the nipple to fill up the mammary fat pad at the age of 3-12 weeks. The epithelial lining consists of luminal cells that produce milk, and myoepithelial cells that are responsible for contraction during lactation [4]. Different theories regarding the mammary stem cell exist. One model proposes the existence of basal stem cells with the property of generating both luminal and basal/myoepithelial lineages [6, 7]. Another hypothesis is that luminal and basal/myoepithelial are generated by a committed lineage-restricted progenitor cell [8]. Although controversy still surrounds the field, current evidence points to a scenario whereby a luminal progenitor may be at the origin of basal-like BRCA-associated breast cancer [9, 10].

Standard treatment of *BRCA1*-associated breast cancer does not differ from other types of breast cancer, although targeted approaches are emerging. However, because BRCA-deficient cells are unable to correct stalled DNA replication forks via homologous repair, these cells are sensitive to platinum-based chemotherapeutics that arrest replication [11-15]. Accordingly, BRCA-deficient cells are more sensitive to poly (ADP)-ribose polymerase 1 (PARP1) inhibition, which is involved in base excision repair [16]. Clinical trials have already tested the efficacy of pharmacological inhibition of PARP with overall promising results [17-24].

The only effective preventive options for BRCA mutation carriers are currently bilateral prophylactic mastectomy (PM) and prophylactic bilateral salpingo-oophorectomy (PBSO). PM yields a risk reducing effect of more than 90-100% in healthy *BRCA1* and *BRCA2* mutation carriers [25-27] and PBSO decreases breast cancer risk in BRCA mutation carriers without prior breast cancer approximately by 50% [26, 28, 29]. An alternative and less invasive preventive strategy option is the use of hormonal therapy as chemo prevention. Inhibition of estrogen receptor function decreases breast cancer risk in healthy *BRCA2* mutation carriers, but not in *BRCA1* mutation carriers [30]. Alternatively, cancer-related mortality in BRCA mutation carriers may be managed through intensive surveillance using mammography and MRI, or biomarkers in nipple fluid like methylation [31]. Efficiency of this approach however is limited because of high breast tissue density in young women and the aggressive, fast developing nature of BRCA-associated breast tumors. Annual MRI detects the majority of breast cancers at an early and favorable stage [32, 33], but a drawback of MRI is the higher rate of false-positive results leading to biopsies of non-diseased tissue and distress [34-37].

An alternative and attractive way to prevent breast cancer development in BRCA mutation carriers may be the local administration of ablative agents to the mammary ductal system via the nipple. Preclinical studies in which chemotherapeutics were used intraductally (ID), such as pegylated liposomal doxorubicin (PLD), 5-fluorouracil, carboplatin, methotrexate, and paclitaxel, show promising results [38-40]. ID chemotherapy was also explored in phase I trials in women with ductal carcinoma *in situ* (DCIS) and invasive breast cancer prior to mastectomy, where ID administration of PLD and cisplatin was well tolerated with mild adverse events. Moreover, pathological changes could be found in the treated ducts [40-42].

Here, we explored the efficacy and safety of ID cisplatin treatment as an alternative prophylactic therapy in the prevention of *BRCA1*-associated breast cancer formation, in combination with PARP-inhibition.

## RESULTS

### Intraductal cisplatin reduces repopulating capacity of the mammary gland

**Figure 1:**
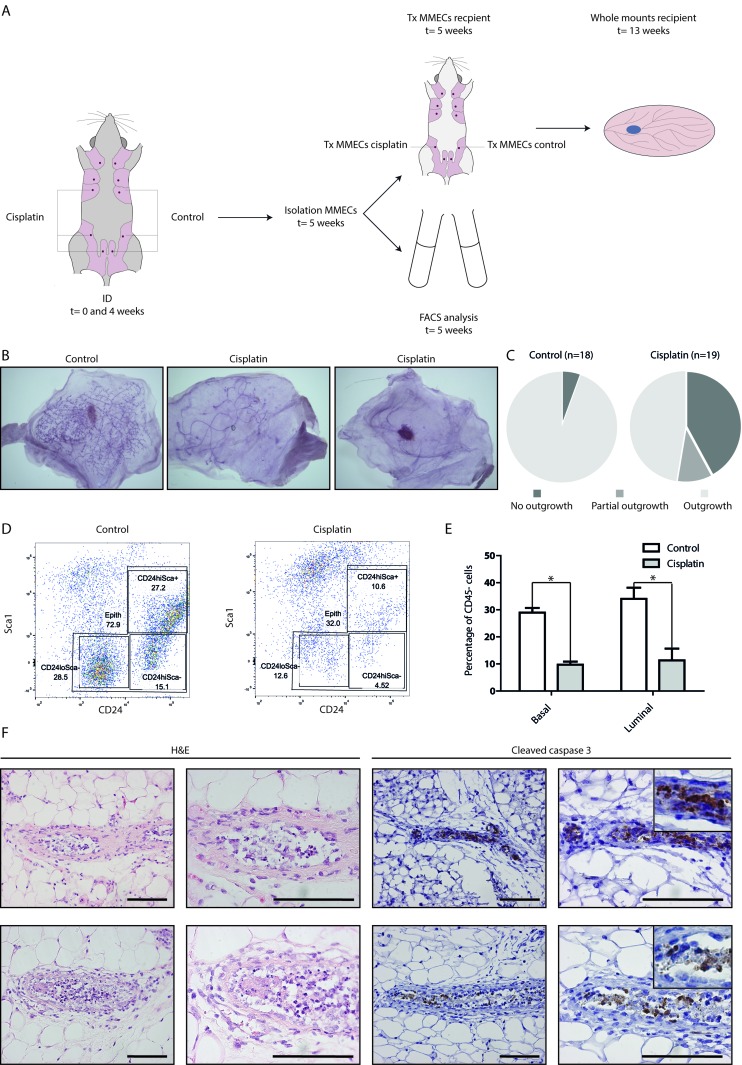
ID cisplatin treatment reduces epithelial viability and leads to an inhibition of mammary gland repopulation **A.** Experimental set-up. Post-breeder mice were subjected to 2 ID cisplatin treatments with a 4-week interval in the right-sided glands number 3, 4, and 5. The contralateral corresponding mammary glands served as control. Next, MMECs were harvested and transplanted into syngeneic mice (upper part) or analyzed by FACS (lower part). **B.** and **C.** Cisplatin inhibits the mammary repopulating capacity. Depicted are representative samples of whole mount analyses, showing complete outgrowth after control treatment (B; left), rudimentary outgrowth (B; middle), or no outgrowth (B; right) after ID cisplatin treatment. Quantifications of the control (left pie chart) and ID cisplatin treated glands (right pie chart) are shown in (C). **D.** and **E.** ID cisplatin treatment leads to an overall reduction of MMECs. Representative plots of the basal and luminal cell populations using FACS analysis after control (D; left dot plot), or ID cisplatin treatment (D; right dot plot). Quantifications of basal and luminal MMECs are shown in (E). Basal MMECs were defined as DAPI-;CD45-;Sca-;CD24+/low and luminal MMECs as DAPI-;CD45-;Sca-or+;CD24+/hi. Shown is an average of 9 mice per condition. Error bars represent SEM; * = *p* < 0.05. **F.** Cisplatin-treated glands showed morphological changes matching apoptosis and an induction of cleaved caspase 3 expression in the epithelial lining of the mammary duct. Representative pictures of H&E and cleaved caspase 3 stainings of mammary gland tissue slides after ID cisplatin treatment are shown. Scale bar is 100 μm, upper right pictures are blow-ups of caspase 3 positive cells.

To test the effect of ID administered platinum on ductal outgrowth, we injected post-breeder wild type mice (ranging in age between 12-30 weeks; primiparous; average litter size of 7; mean time post weaning of 42 days) twice with ID cisplatin in gland number 3, 4, and 5 on the right side and with control on the left side with a 4-week interval (Figure 1A). One week after the second treatment, we isolated mouse mammary epithelial cells (MMECs) from the 3^rd^ and 4^th^ mammary glands that were injected into the cleared mammary fat pad of 3-week old syngeneic recipient mice. ID cisplatin significantly lowered outgrowth after transplantation compared to control injection (*p* = 0.010; Figure 1B; 1C). Whole mount analysis showed ductal outgrowth in 17 of 18 (94.4%) transplantations of the control-treated mammary glands and in 11 of 19 transplantations in the cisplatin-treated glands (57.9%), of which 2 showed only rudimentary duct formation.

Next, we performed FACS analysis, which indicated that ID administered cisplatin induced a significant and uniform reduction in the basal and luminal populations (*p* = 0.008 compared to control for luminal, and *p* < 0.001 for basal population in *n* = 9 analyzed glands; Figure 1D; 1E). The effect of cisplatin appeared to specifically reduce the epithelial mammary cells, since the number of non-epithelial cells was not decreased proportionally after ID treatment (Figure 1D; upper left quadrant). Further, we observed that cisplatin-treated glands showed an induction of cleaved caspase 3 expression in the epithelial lining of the mammary duct (Figure 1F). We therefore conclude that ID cisplatin treatment reduces the repopulating capacity of the wild type mammary gland.

### Intraductal cisplatin impairs lobulo-alveolar development

**Figure 2:**
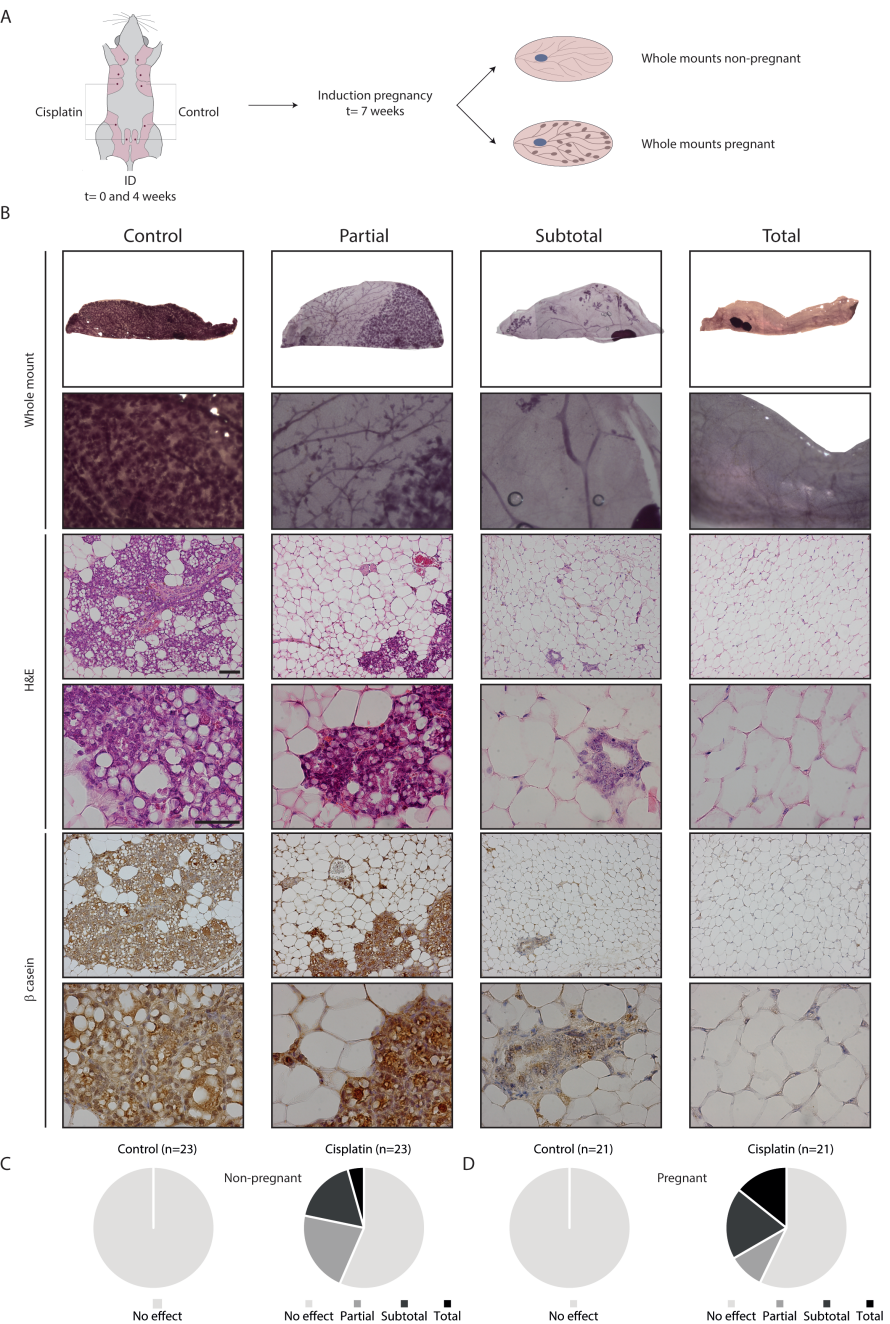
Functional effect of ID cisplatin on the mammary gland and pregnancy-induced lobulo-alveolar development **A.** Experimental set-up. Post-breeder mice were subjected to two ID cisplatin treatments with a 4-week interval in the right-sided glands number 3, 4, and 5. The contralateral corresponding mammary glands served as control. Next, whole mounts were analyzed at day 17.5 of pregnancy or at a similar time point in non-pregnant mice. **B.** Representative pictures showing no effect in control (first column) or partial (second column), subtotal (third column), or total effect (fourth column) after ID cisplatin treatment. The top two rows show whole mount analyses, followed by H&E stainings, and β-casein IHC. Scale bar is 100 μm in lower magnifications and 50 μm in higher magnifications. **C.** ID cisplatin reduces ductal outgrowth in non-pregnant mice (*n* = 23). Quantifications of mammary ductal outgrowth after control (left chart) or ID cisplatin treatment (right chart). **D.** ID cisplatin reduces lobulo-alveolar development in pregnant mice (*n* = 21). Quantifications of mammary ductal outgrowth after control (left chart) or ID cisplatin treatment (right chart).

To assess the effect of treatment on pregnancy-associated lobulo-alveolar development, we analyzed mammary gland whole mounts after ID treatment of both non-pregnant and pregnant mice (Figure 2A). ID cisplatin reduced ductulo-lobular outgrowth in both non-pregnant and pregnant mice. Representative whole mounts and corresponding H&E and β-casein stainings are shown in Figure 2B. In all control mammary glands from both non-pregnant and pregnant mice, ductal outgrowth and alveolar differentiation was uniform and complete. ID cisplatin showed no effect in 13 glands of 23 non-pregnant mice (56.6%), partial effect in 5 (21.7%), subtotal in 4 (17.4%), and total effect in 1 (4.3%) (Figure 2C). In pregnant mice ID cisplatin did not lead to an inhibition of alveolar development in 12 of 21 mice (57.1%), while we observed a partial response in 2 (9.5%), subtotal in 4 (19.0%), and total response in 3 (14.3%) glands (Figure 2D). In summary, ID cisplatin administration induced a significant reduction in ductal outgrowth (defined as (sub) total effect) in both non-pregnant and pregnant mice (*p* = 0.018 and 0.004, respectively).

### Intraductal cisplatin and olaparib increase tumor-free latency in a mouse model of *BRCA1*-associated human breast cancer

**Figure 3:**
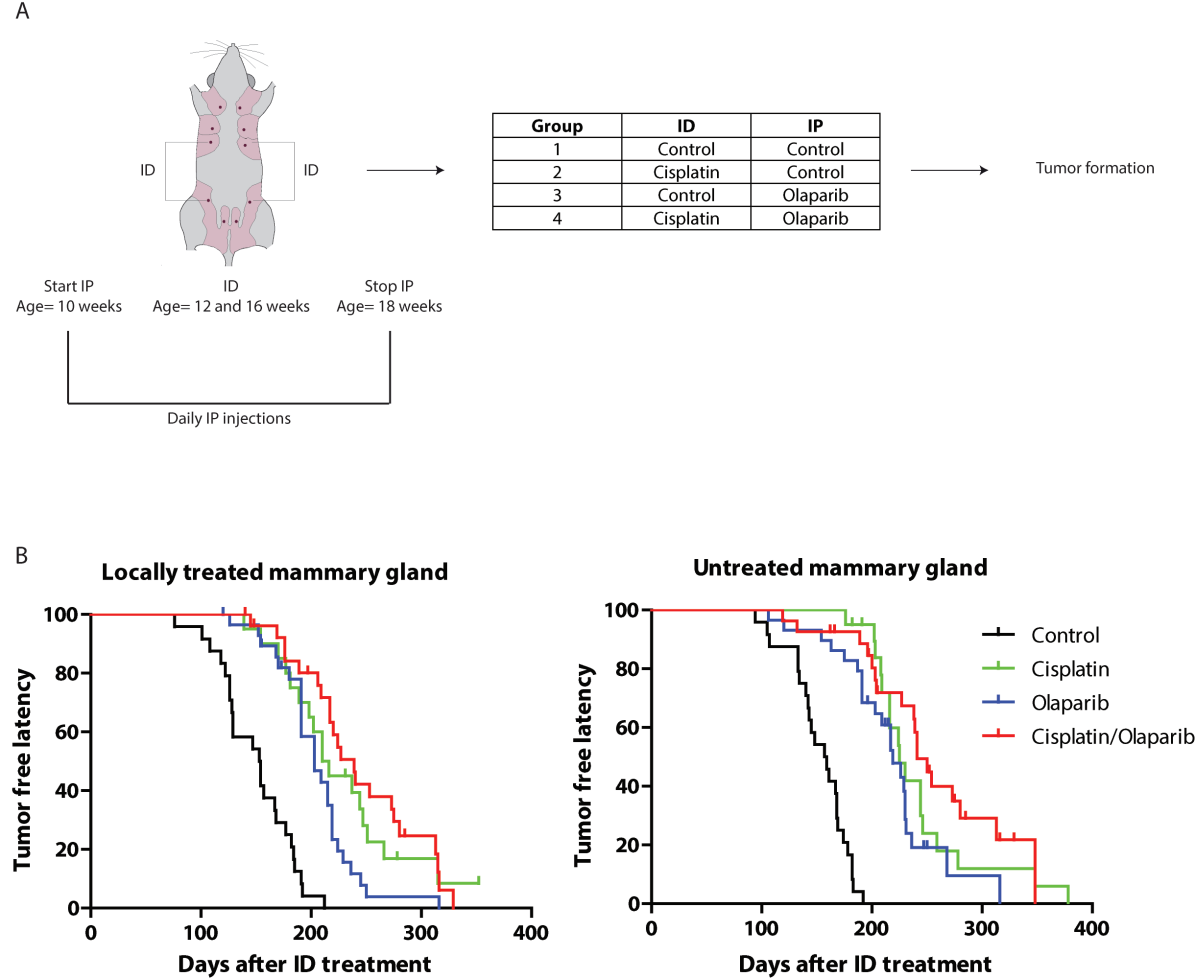
Combined prophylactic cisplatin and olaparib treatment in a *WAPCre;Brca1F/F;Trp53F/F* sporadic mouse model leads to a later onset of breast tumor formation **A.** Experimental set-up. Virgin *WAPCre;Brca1F/F;Trp53F/F* were treated twice in glands number 3 and 4 with either control or cisplatin at the age of 12 and 16 weeks. During the ID treatments, mice were IP injected with control or olaparib on a daily basis from the age of 10 to 18 weeks. In total 4 treatment groups were analyzed, i.e. control (*n* = 24), ID cisplatin monotherapy (*n* = 20), IP olaparib monotherapy (*n* = 29) and ID cisplatin combined with IP olaparib combination therapy (*n* = 27). **B.** Kaplan-Meier curves showing the tumor-free latency post treatment of mammary glands tumor in the treated (glands number 3 and 4; left graph) and untreated mammary glands (glands number 1, 2, and 5; right graph).

We next investigated the preventive effect of ID cisplatin in a mouse model for *BRCA1*-associated human breast cancer. Since tumors in a K14cre-driven variant of this model were highly sensitive to a combination of cisplatin and the PARP-inhibitor olaparib [14], we tested both prophylactic monotherapies and the combination of ID cisplatin and olaparib in the whey acidic protein (WAP)cre-driven tumor model. We chose the WAPcre model for our studies because the overt induction of skin tumors in the K14cre models interferes with the analysis of mammary tumor formation [43]. Tumor latency and incidence in the *WAPCre;Brca1F/F;Trp53F/F* model are shown in Supplementary Figure S1 and PCR results of tumors in Supplemental Figure 2. Female virgin *WAPcre;Brca1F/F;Trp53F/F* mice were injected twice in glands 3 and 4. Either all third and fourth glands were treated with control or all 4 treated glands were injected with cisplatin at the age of 12 and 16 weeks. Prior, during and post ID cisplatin treatment, mice were daily injected intraperitoneally (IP) with control or olaparib and followed for tumor formation (Figure 3A). ID cisplatin monotherapy significantly increased the median tumor-free latency (T50) in ID injected glands from 153 days in control to 210 days in cisplatin treated mice (*p* < 0.0001; Figure 3B). IP treatment using olaparib monotherapy also led to an increase in tumor-free latency (T50 = 203 versus 153 days; *p* < 0.001; Figure 3B). Moreover, using a combination therapy of ID cisplatin and IP olaparib, we observed an increase in T50 from 153 to 239 days (*p* < 0.001; Figure 3B). Dual therapy using ID cisplatin and olaparib led to a longer tumor-free latency compared to olaparib monotherapy (*p* = 0.004) but not compared to cisplatin monotherapy (*p* = 0.506).

Strikingly, similar effects were obtained when analyzing untreated mammary glands, i.e. glands number 1, 2 and 5. Here, the T50 increased significantly from 157 days in control to 225 days in cisplatin treated animals (*p* < 0.0001; Figure 3B). Olaparib induced an increase in T50 to 219 days (*p* < 0.0001), and combination treatment led to T50 of 241 days (*p* < 0.0001; Figure 3B), suggesting that local ID treatment results in systemic exposure and subsequent inhibition of tumor development.

ID cisplatin reduced tumor incidence in the locally treated gland to 85.0% (*p* = 0.086 compared to control). For olaparib this was 89.7% (*p* = 0.242), and in the combination group tumor incidence was 81.5% (*p* = 0.052). Monotherapy with cisplatin ID did not significantly differ from olaparib monotherapy regarding tumor incidence (*p* = 0.677). Also, a dual ID cisplatin and olaparib treatment was not significantly better than either monotherapies. Tumor incidence of the untreated glands decreased to 85.0% in cisplatin (*p* = 0.086), 75.9% in olaparib (*p* = 0.012), and 66.7% in the combination treatment group (*p* = 0.002). Metastases formation did not significantly change between different treatment groups (Supplementary Table S1).

In short, a combination of ID cisplatin with systemic olaparib increases tumor-free latency in a mouse model of Brca1-associated breast cancer. However, ID injection leads to systemic exposure of cisplatin.

### Pharmacokinetics of intraductal cisplatin

**Figure 4:**
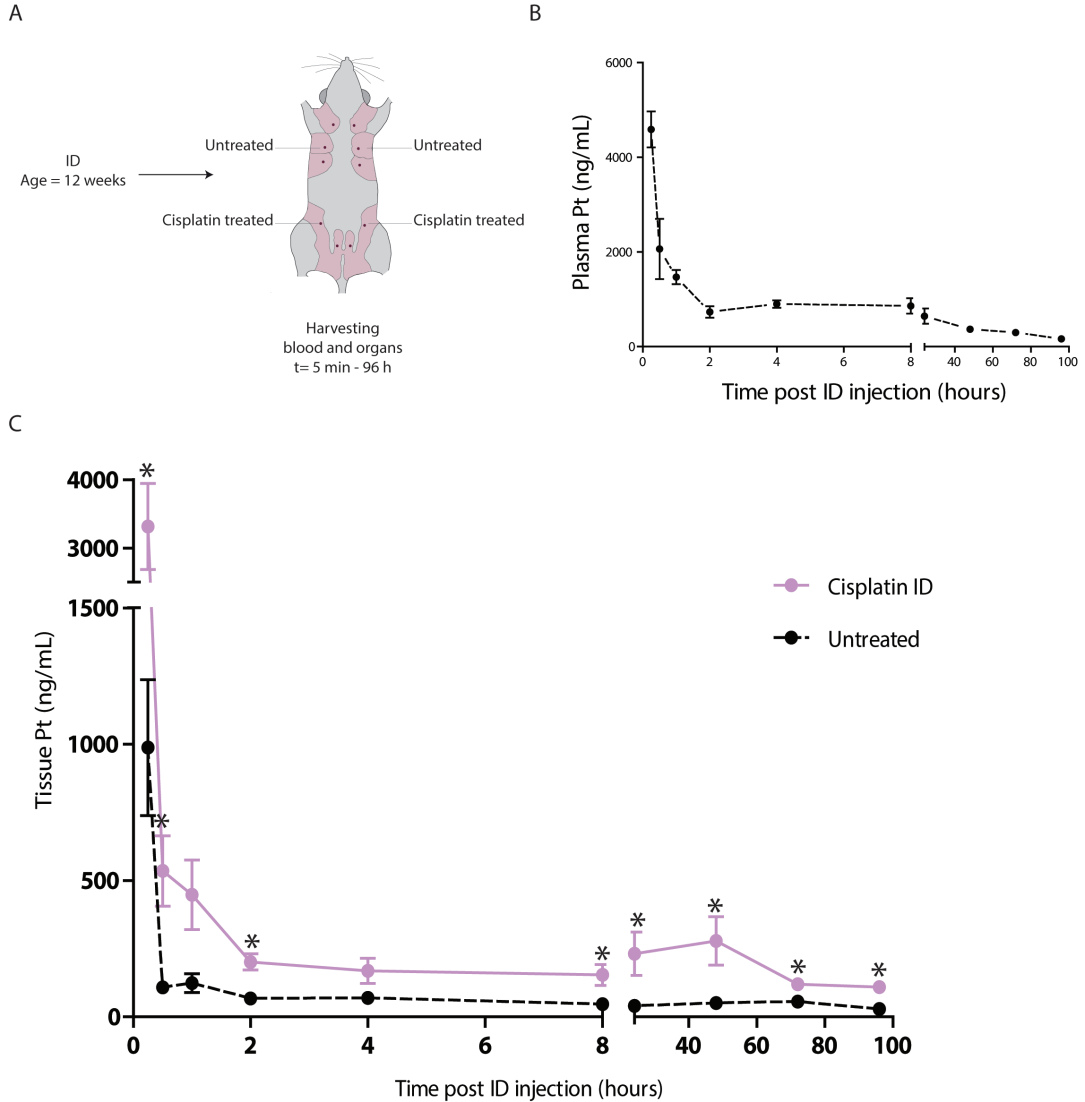
Pharmacokinetics of the ID cisplatin treatment **A.** Experimental set-up. Virgin wild type mice were treated with ID cisplatin in glands number 3 and 4 at the age of 12 weeks. Mice were sacrificed at different time points after ID injections (see text for details, *n* = 3 mice per time point) and plasma and mammary gland tissue samples were harvested. **B.** Plasma platinum concentrations after ID treatment. Shown are the average concentrations per time point with SEM. **C.** Mammary gland platinum concentrations after ID cisplatin treatment. The black line shows the concentrations in untreated glands (gland number 2). The pink line depicts the concentrations in the treated mammary glands (gland number 4). Shown are the average concentrations per time point with SEM; * = *p* < 0.05.

To determine the extent of systemic exposure to platinum after ID injection, we harvested plasma samples after ID treatment with cisplatin injection of the 3^rd^ and 4^th^ mammary glands of 12 week old wild type mice (Figure 4A). Highest plasma concentrations were measured 15 minutes after treatment with an average of 4.6 μg/ml (Figure 4B). After 5 minutes, platinum was detected in plasma with a mean concentration of 1.0 μg/ml when injecting one mammary gland. After 96 hours the AUC value was 45.6 μg•ml/h, comparable to a reported AUC of 47.9 μg•ml/h after intra-tumoral (IT) injection of a similar dose of cisplatin [44].

We also determined platinum tissue concentrations in locally treated and distant untreated mammary glands (4^th^ and 2^nd^ mammary glands, respectively, see Figure 4C). Concentrations peaked at 15 minutes with a mean platinum concentration of 3.32 μg/ml in locally treated versus 0.99 μg/ml in the untreated glands. Platinum concentrations in the untreated glands were significantly lower than in locally treated glands (*p* = 0.015). After 96 hours the AUC value of locally treated glands was 18.75 μg•ml/h compared to 4.73 μg•ml/h in systemically treated glands, which is 25.2% of the locally exposed glands.

### Long-term local and systemic effects of intraductal cisplatin

**Figure 5:**
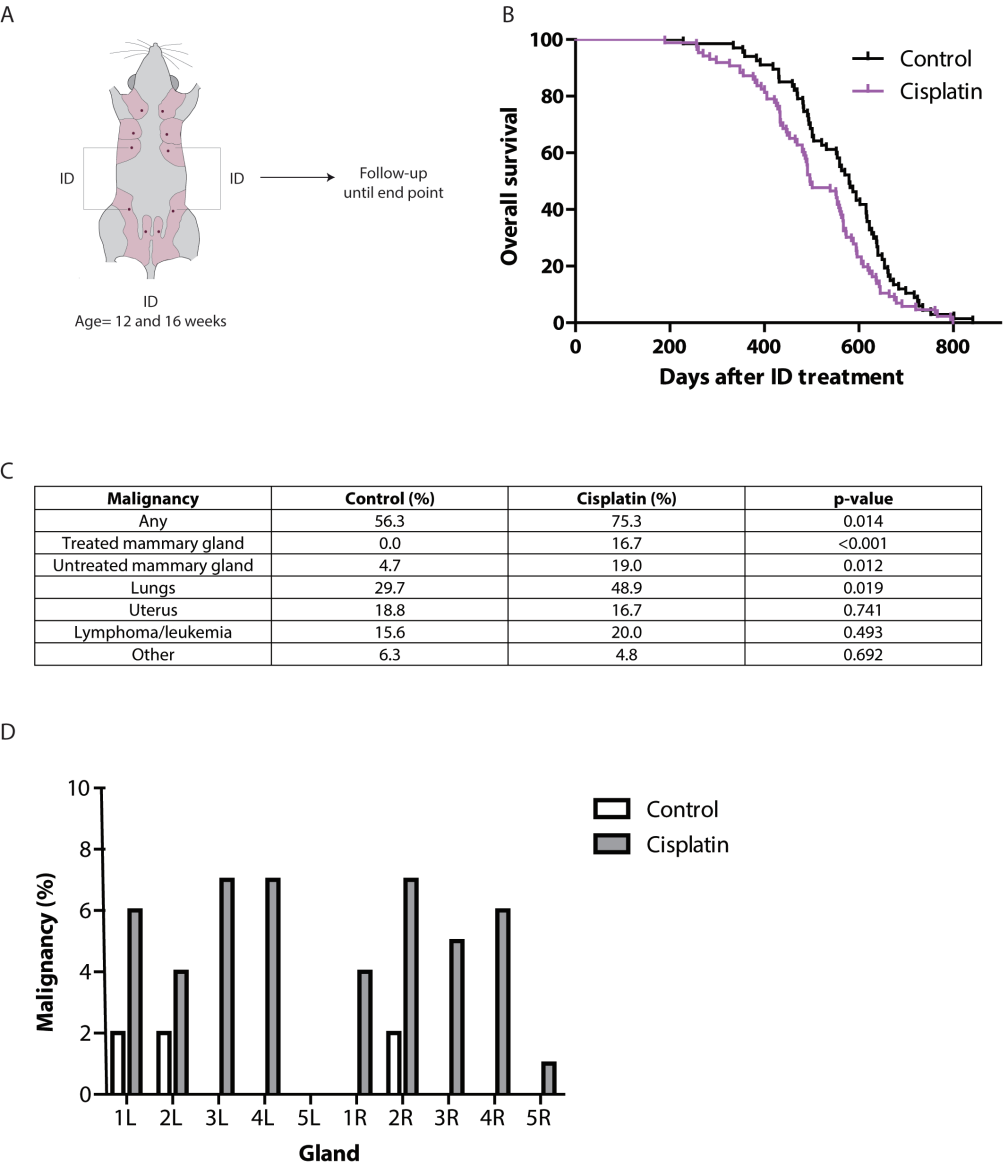
Long-term effects of ID cisplatin treatment **A.** Experimental-set up. Virgin F1 (FVB/N;Ola129Hsd) mice were treated twice in glands 3 and 4 with either cisplatin or control at the age of 12 and 16 weeks. Next, mice were followed in time for tumor development. **B.** Kaplan-Meier curves showing overall survival of control (black line; *n* = 67) or ID cisplatin (pink line; n = 86) treated mice. **C.** Table showing the incidence of malignancies in control and ID cisplatin treated mice. The category ‘other tumors’ consisted of sarcoma of unknown origin, hemangiosarcoma of the spleen, carcinoma of the oral cavity, and squamous carcinoma in the anogenital region in control mice. In ID cisplatin treated mice ‘other tumors’ comprised hepatocellular carcinoma (*n* = 2), carcinoma of the salivary gland, and angiosarcoma in the mammary gland. **D.** Bar graph showing the breast tumor incidence in control and ID cisplatin treated mice per mammary gland.

To serve as an alternative prophylaxis in young BRCA-mutation carriers, long-term carcinogenicity of ID cisplatin must be minimal. To test this, we ID injected the 3^rd^ and 4^th^ mammary glands of post-pubertal wild type mice at the age of 12 and 16 weeks with either control (*n* = 67) or cisplatin (*n* = 86), which were followed longitudinally until death or disease (Figure 5A). Due to early death caused by mammary tumor formation in the mouse model carrying conditional homozygous Trp53 alleles we, used allogeneic wild-type mice for long-term carcinogenicity studies. Although ID treatment of cisplatin induced a shorter tumor-free latency based on histopathology data, the reduction in latency was only observed in aged (>560 days) mice (Figure 5B). Also, we observed an effect on overall survival when comparing treated *versus* control female mice (*p* = 0.019; Figure 5B). Mammary tumors developed more often in the ID cisplatin treated mammary glands (21 of 336 locally treated glands (6.3%)) than in control mice (0 of 252 locally treated glands; *p* < 0.001; Figure 5C and 5D), albeit in aged female mice (679 days after the second ID injection), which did not lead to an increase metastatic dissemination. Importantly, tumor incidence in distant untreated glands was increased. Cisplatin treated mice developed breast tumors in untreated glands in 3.6% (18 of 504 glands) compared to 0.8% in control mice (3 of 378 glands; *p* = 0.007; Figure 5C and 5D). Finally, primary adenocarcinomas of the lung were found in 48.9% in cisplatin versus 29.7% in control treated mice (*p* = 0.019; Figure 5C). The incidence of gynecological, lymphoproliferative and other tumors did not significantly differ between both treatment groups (*p* = 0.741, 0.493 and 0.692, respectively; Figure 5C). Benign pathology of the aged female mice is shown in Supplementary Table S2.

Given the increase in both local and systemic tumor incidence in mice, we conclude that ID administration of cisplatin under these conditions cannot upfront be regarded as a safe alternative prophylactic therapy to prevent *BRCA1*-associated breast cancer formation in humans.

## DISCUSSION

ID cisplatin treatment leads to a uniform reduction of the epithelial mammary cells and thereby prolongs breast tumor-free latency in a conditional *BRCA1*-associated mouse model. However, our results also show that the success rate of ID treatment using cisplatin is variable. Multiple functional assays, including whole mount and transplantation assays, resulted in a successful ablation in approximately half of the recipient mice. This partial effect can be explained by *e.g.* incomplete injection penetrance, whereby not all ductal branches have been exposed to cisplatin. Moreover, the progenitor cell leading to cancer is presumably a low proliferating cell and as such less susceptible to platinum-based drugs. Another explanation could be the that proliferative differences due to estrus cycling may influence the effectiveness of cisplatin [45, 46], which suggests that ID treatment should be tailored to specific estrus stages for successful cisplatin-based prophylaxis.

To our surprise, ID cisplatin prolonged tumor-free latency in untreated mammary glands. Since Brca1 inactivation and subsequent tumor formation in the WAPcre-driven model occurs during the first estrus cycle (independent of pregnancy-induced WAP activation) [43], we anticipate that this is probably due to the fact that prior to treatment, *WAPcre;Brca1F/F;Trp53F/F* female mice contain Brca1-deficient mammary epithelial cells. Also, because Brca-/- mouse embryonic stem cell lines are 5-fold more sensitive to cisplatin than Brca+/+ wild type cells [11], it is likely that Brca1 loss underpins the observed sensitivity to cisplatin. Although rapid systemic distribution of platinum drugs is evident after ID treatment (our data and [41]), we also showed that platinum levels in the circulation and untreated glands were lower than can be expected after systemic treatment [44]. A study using intra tumor (IT) injection of cisplatin and found a comparable AUC to the value we found after ID injection. Compared to IP injection, IT administration led to lower plasma concentrations. Systemic exposure to platinum after IT injection was calculated to be 65-75% of the exposure after systemic administration. Since IT and ID cisplatin administration showed a similar pharmacokinetic profile, we expect that also ID cisplatin will lead to lower systemic platinum levels compared to systemic treatment [44]. Our findings are in agreement with studies showing that ID treatment with other chemotherapeutics also leads to systemic exposure [39, 40].

Long-term carcinogenicity is a major point of concern in prophylactic treatments using chemotherapeutics. The same cytotoxic agent can be used to treat cancer, but can also drive carcinogenesis [47]. ID PLD is reported to induce malignant mammary gland tumors in wild type mice after a follow-up of 42 weeks [48]. When MMECs were isolated from mice treated with ID PLD, recipients developed tumors [48]. Our current study shows that cisplatin leads to tumor formation in 6.3% of treated mammary glands more than 95 weeks post treatment. The induction of late tumors also affected overall survival after cisplatin treatment. The induction of secondary malignancies may be explained by platinum release from regenerating tissue leading to circulating plasma platinum levels up to 20 years after treatment [49, 50]. Ovarian cancer survivors show an increased breast cancer risk, however the influence of chemotherapy was either not investigated or not convincingly shown [51, 52]. Genetic and reproductive factors predisposing to ovarian cancer may also have contributed to breast cancer development [52]. In contrast to our findings in mice, it was shown that long-term testicular and ovarian cancer survivors have no increased lung cancer risk [51-53], and testicular cancer patients treated with chemotherapy only do not develop more lung tumors during follow-up [54]. Despite the obvious species differences in rodents and humans that may underlie the long-term tumor formation after intraductal cisplatin in our used preclinical models, we think that ID administration of solvable cisplatin cannot upfront be regarded as a safe alternative to prevent *BRCA*-associated breast cancer in genetic carriers. Possible alternative ways of delivering an immobilized yet locally active chemotherapy should be developed to prevent systemic exposure. Since intraductal PLD also induced mammary tumors, we feel that this precludes the use of ID chemotherapy in general for preventive purposes. Using local therapy intraductally however seems to be feasible and effective and could be combined with the use of nanocarriers [55], liposomes [56], or antidotes [57].

Olaparib was already shown to be effective in breast cancer treatment [17-21, 23, 24], but now we also demonstrate the prophylactic effect of systemic PARP-inhibition in *Brca1*-associated breast cancer. Combining ID cisplatin and olaparib led to an additional effect in breast tumor incidence compared to both monotherapies without increasing toxicity. Future studies are needed to investigate the optimal dosages of combination treatment and the long-term safety of olaparib treatment.

In conclusion, we demonstrate that although ID treatment using cisplatin, especially in combination with PARP-inhibition successfully attenuates tumor formation in a clinically relevant mouse model of *BRCA1*-associated breast cancer, it leads to long-term local and systemic secondary malignancies in mice and may not be safe in humans.

## MATERIALS AND METHODS

### Mice

F1 Wild type mice were derived from crossings between FVB/NCrl (Charles River) and 129P2/OlaHsd (Harlan). *WAPcre;BrcaF/F;Trp53F/F* mice were generated by crossing Brca1F/F conditional female mice [58] with male *WAPcre;Trp53F/F* mice [43]. Female mice in a model using WAPCre as a promoter, develop mammary tumors for which the onset, incidence and metastasis are pregnancy and lactation-independent [43]. Genotyping and deletion status of the Trp53^F^, Trp53^Δ^, Brca1^F^, Brca1^Δ^ and WAPcre alleles was done as described [43, 58].

Onset of tumor growth was monitored twice weekly from the age of 4 months onward. Mammary tumor volume was determined by caliper measurements using the following formula: 4/3*((3.14)*((length+width)/4)^3). Animals were euthanized if mammary tumors reached a size of 1000 mm^3^ or in case of severe discomfort otherwise. Animals were scored as having a tumor when there was a tumor palpable or based on post mortem histological assessment. Tumor-free latency was calculated as the number of days after the second ID injection. All animal experiments were performed according to institutional guidelines and national regulations (DEC2010.III.08.102, 2011.III.04.050, 2012.III.01.005, 2012.III.09.086).

### Drugs

Cisplatin (1 mg/mL in saline, Pharmachemie B.V.) was administered ID in a total volume of 50 μl per mammary gland. To visualize extravasation or incorrect injection, cisplatin or control was mixed with 0.05% methylene blue. Olaparib was synthesized by Syncom with a purity of more than 99% by HPLC. Olaparib was used by diluting 50 mg/ml stocks in DMSO with 10% 2-hydroxyl-propyl-β-cyclodextrine/PBS such that the final volume administered intraperitoneally (IP) was 10 μL/g of body weight. Saline 0.9% (Fresenius Kabi) was used as control.

### Intraductal injection

Mice were anesthetized by isoflurane and 100 μl (0.03 mg/ml) Temgesic (buprenorphine) was injected subcutaneously (SC) as analgesic treatment. Mammary ducts were cannulated using a 1.0-cm, 33G, blunt-ended needle (Hamilton, 66780305) attached to a 50 μl glass syringe (Hamilton 66765501). Drugs or control were injected into the mammary gland while visualizing the nipple opening under a dissection microscope [39].

### Orthotopic transplantation MMECs

Primary mouse mammary epithelium cells (MMECs) were obtained from mammary glands after removal of the intra-mammary lymph nodes. Tissue was finely chopped three times using a McIlwain tissue chopper (The Mickle Laboratory Engineering Co.) and digested for 1 hr at 37°C in serum-free DMEM-F12 medium (Invitrogen Life Technologies) containing 0.1 mg/ml porcine pancreatic trypsin (Gibco) and 0.2 mg/ml collagenase A (Roche). Cells were washed and fibroblasts were allowed to adhere for 1 hr at 37°C. Non-adherent epithelial cells were collected, washed, pelleted in 40 μl serum-free DMEM-F12 and kept on ice until transplantation.

MMECs were transplanted in 3-week-old wild type syngeneic female recipient mice. Recipient mice were anesthetized by isoflurane. Temgesic (buprenorphine; 100 μl (0.03 mg/ml)) was injected SC as analgesic treatment. The fourth (inguinal) mammary gland was exposed and endogenous mammary epithelial tissue was removed. Next, MMECs were injected in the cleared fat pad using a 50 μl Hamilton syringe, after which the animals were sutured. Whole mount analysis was performed 8 weeks after transplantation.

### FACS analysis

After enzymatic digestion of the mammary tissue, cells were incubated with red blood cell lysis buffer (8.26 g NH_4_Cl, 1 g NaHCO_3_, 0.0378 g EDTA in 1 L dH_2_0) to remove erythrocytes (15 minutes). Cells were washed and fibroblasts were allowed to adhere for 1 hr at 37°C. Non-adherent epithelial cells were collected, washed in 0.02% EDTA-PBS solution, pelleted and resuspended in 0.25% w/v trypsin/0.2% w/v EDTA in PBS for incubation at 37°C for 2 minutes. Cells were dissociated through a 70 μm cell strainer and resuspended in fresh RPMI/10% FCS for quantification. Cells were incubated with primary antibodies for 45 minutes on ice (FITC-conjugated anti-CD24 (clone M1/69, BD Biosciences, 0.5 μg/ml), PE-Cy5-conjugated anti-CD45 (clone 30-F11, BD Biosciences, 0.25 μg/ml), and PE-conjugated anti-Sca-1 (clone D7, 0.5 μg/ml; BD Biosciences, 0.5 μg/ml)). Before FACS analysis, cells were incubated with 0.01% 4’, 6-diamidino-2-phenylindole dihydrochloride (DAPI) for 15 minutes. Analyses were carried out on a BD FACSVantageSE DiVa (BD Biosciences) and gating was performed as described [59]. Flow Jo software version 10 was used for analysis.

### Whole mount analysis

Mammary glands were dissected and stretched on a glass slide. Glands were fixed in a mixture of methanol:1, 1, 1-trichloroethane:acetic acid (6:3:1) for 4 hours and stained overnight with carmine aluminium staining solution (2 g/l carmine, 5 g/l aluminium potassium sulphate dissolved in water). After stepwise dehydration in 70%, 95% and 100% ethanol, glands were cleared in xylene for 10 minutes and photographs were taken with a Zeiss Stemi 2000-C stereo microscope. We defined effect size as follows: total effect (0% outgrowth), subtotal (up to 25% outgrowth), partial (more than 25% outgrowth), and no effect (100% outgrowth).

### Histological analysis

Tissues were isolated and fixed in 4% formaldehyde. Tissues were dehydrated, cut into 4 μm sections, and stained with hematoxylin and eosin. For immunohistochemical stainings, fixed sections were rehydrated and incubated with primary antibodies against β-casein (Santa-Cruz SC-30042, FL-231, rabbit, 1:100) and cleaved caspase 3 (Cell Signaling 9661, rabbit, 1:500). Endogenous peroxidases were blocked with 3% H_2_O_2_ and biotin-conjugated secondary antibodies were used, followed by incubation with HRP-conjugated streptavidin-biotin complex (DAKO). Substrate was developed with DAB (DAKO) and pictures were made using a Nikon Eclipse E800 with a Nikon digital camera DXM1200. Appropriate positive and negative controls were used throughout.

### Pharmacokinetic analysis

Mice were euthanized at 5 minutes, 15 minutes, 30 minutes, 1, 2, 4, 8, 24, 48, 72 and 96 hour intervals after ID injection. Blood was collected via cardiac puncture and tissue was isolated and frozen. Platinum concentrations were measured by a validated Inductively Coupled Plasma Mass Spectrometry (ICPMS) method as described by Brouwers *et al.* [60].

### Statistical analysis

For statistical analyses IBM SPSS Statistics Version 20 was used. For dichotomous variables Chi square tests were used; for continuous variables t-test (normal distribution), Mann Whitney test (no normal distribution, no paired data) or Wilcoxon signed rank test (no normal distribution, paired data). For survival analysis Kaplan-Meier curves and Log-rank (Mantel-Cox) tests were performed. A two-sided *p*-value <0.05 was considered statistically significant.

### SUPPLEMENTARY MATERIALS FIGURES AND TABLES





## References

[1] Njiaju UO, Olopade OI (2012). Genetic determinants of breast cancer risk: a review of current literature and issues pertaining to clinical application. Breast J.

[2] Couch FJ, Wang X, McGuffog L, Lee A, Olswold C, Kuchenbaecker KB, Soucy P, Fredericksen Z, Barrowdale D, Dennis J, Gaudet MM, Dicks E, Kosel M (2013). Genome-wide association study in BRCA1 mutation carriers identifies novel loci associated with breast and ovarian cancer risk. PLoS Genet.

[3] Gaudet MM, Kuchenbaecker KB, Vijai J, Klein RJ, Kirchhoff T, McGuffog L, Barrowdale D, Dunning AM, Lee A, Dennis J, Healey S, Dicks E, Soucy P (2013). Identification of a BRCA2-specific modifier locus at 6p24 related to breast cancer risk. PLoS Genet.

[4] Buckley NE, Mullan PB (2012). BRCA1--conductor of the breast stem cell orchestra: the role of BRCA1 in mammary gland development and identification of cell of origin of BRCA1 mutant breast cancer. Stem Cell Rev.

[5] Visvader JE, Stingl J (2014). Mammary stem cells and the differentiation hierarchy: current status and perspectives. Genes Dev.

[6] Rios AC, Fu NY, Lindeman GJ, Visvader JE (2014). In situ identification of bipotent stem cells in the mammary gland. Nature.

[7] Prater MD, Petit V, Russell IA, Giraddi RR, Shehata M, Menon S, Schulte R, Kalajzic I, Rath N, Olson MF, Metzger D, Faraldo MM, Deugnier MA (2014). Mammary stem cells have myoepithelial cell properties. Nat Cell Biol.

[8] Keymeulen A Van, Rocha AS, Ousset M, Beck B, Bouvencourt G, Rock J, Sharma N, Dekoninck S, Blanpain C (2011). Distinct stem cells contribute to mammary gland development and maintenance. Nature.

[9] Lim E, Vaillant F, Wu D, Forrest NC, Pal B, Hart AH, Asselin-Labat ML, Gyorki DE, Ward T, Partanen A, Feleppa F, Huschtscha LI, Thorne HJ (2009). Aberrant luminal progenitors as the candidate target population for basal tumor development in BRCA1 mutation carriers. Nat Med.

[10] Molyneux G, Geyer FC, Magnay FA, McCarthy A, Kendrick H, Natrajan R, Mackay A, Grigoriadis A, Tutt A, Ashworth A, Reis-Filho JS, Smalley MJ (2010). BRCA1 basal-like breast cancers originate from luminal epithelial progenitors and not from basal stem cells. Cell Stem Cell.

[11] Bhattacharyya A, Ear US, Koller BH, Weichselbaum RR, Bishop DK (2000). The breast cancer susceptibility gene BRCA1 is required for subnuclear assembly of Rad51 and survival following treatment with the DNA cross-linking agent cisplatin. J Biol Chem.

[12] Tassone P, Tagliaferri P, Perricelli A, Blotta S, Quaresima B, Martelli ML, Goel A, Barbieri V, Costanzo F, Boland CR, Venuta S (2003). BRCA1 expression modulates chemosensitivity of BRCA1-defective HCC1937 human breast cancer cells. Br J Cancer.

[13] Sgagias MK, Wagner KU, Hamik B, Stoeger S, Spieker R, Huber LJ, Chodosh LA, Cowan KH (2004). Brca1-deficient murine mammary epithelial cells have increased sensitivity to CDDP and MMS. Cell Cycle.

[14] Rottenberg S, Nygren AO, Pajic M, van Leeuwen FW, van der Heijden I, van de Wetering K, Liu X, de Visser KE, Gilhuijs KG, van Tellingen O, Schouten JP, Jonkers J, Borst P (2007). Selective induction of chemotherapy resistance of mammary tumors in a conditional mouse model for hereditary breast cancer. Proc Natl Acad Sci U S A.

[15] Byrski T, Huzarski T, Dent R, Marczyk E, Jasiowka M, Gronwald J, Jakubowicz J, Cybulski C, Wisniowski R, Godlewski D, Lubinski J, Narod SA (2014). Pathologic complete response to neoadjuvant cisplatin in BRCA1-positive breast cancer patients. Breast Cancer Res Treat.

[16] Farmer H, McCabe N, Lord CJ, Tutt AN, Johnson DA, Richardson TB, Santarosa M, Dillon KJ, Hickson I, Knights C, Martin NM, Jackson SP, Smith GC (2005). Targeting the DNA repair defect in BRCA mutant cells as a therapeutic strategy. Nature.

[17] Fong PC, Boss DS, Yap TA, Tutt A, Wu P, Mergui-Roelvink M, Mortimer P, Swaisland H, Lau A, O’Connor MJ, Ashworth A, Carmichael J, Kaye SB (2009). Inhibition of poly(ADP-ribose) polymerase in tumors from BRCA mutation carriers. N Engl J Med.

[18] Fong PC, Yap TA, Boss DS, Carden CP, Mergui-Roelvink M, Gourley C, De Greve J, Lubinski J, Shanley S, Messiou C, A’Hern R, Tutt A, Ashworth A (2010). Poly(ADP)-ribose polymerase inhibition: frequent durable responses in BRCA carrier ovarian cancer correlating with platinum-free interval. J Clin Oncol.

[19] Audeh MW, Carmichael J, Penson RT, Friedlander M, Powell B, Bell-McGuinn KM, Scott C, Weitzel JN, Oaknin A, Loman N, Lu K, Schmutzler RK, Matulonis U (2010). Oral poly(ADP-ribose) polymerase inhibitor olaparib in patients with BRCA1 or BRCA2 mutations and recurrent ovarian cancer: a proof-of-concept trial. Lancet.

[20] Tutt A, Robson M, Garber JE, Domchek SM, Audeh MW, Weitzel JN, Friedlander M, Arun B, Loman N, Schmutzler RK, Wardley A, Mitchell G, Earl H (2010). Oral poly(ADP-ribose) polymerase inhibitor olaparib in patients with BRCA1 or BRCA2 mutations and advanced breast cancer: a proof-of-concept trial. Lancet.

[21] Gelmon KA, Tischkowitz M, Mackay H, Swenerton K, Robidoux A, Tonkin K, Hirte H, Huntsman D, Clemons M, Gilks B, Yerushalmi R, Macpherson E, Carmichael J (2011). Olaparib in patients with recurrent high-grade serous or poorly differentiated ovarian carcinoma or triple-negative breast cancer: a phase 2, multicentre, open-label, non-randomised study. The LancetOncology.

[22] Thoolen B, Maronpot RR, Harada T, Nyska A, Rousseaux C, Nolte T, Malarkey DE, Kaufmann W, Küttler K, Deschl U, Nakae D, Gregson R, Vinlove MP (2010). Proliferative and nonproliferative lesions of the rat and mouse hepatobiliary system. Toxicol Pathol.

[23] Kaufman B, Shapira-Frommer R, Schmutzler RK, Audeh MW, Friedlander M, Balmaña J, Mitchell G, Fried G, Stemmer SM, Hubert A, Rosengarten O, Steiner M, Loman N (2015). Olaparib monotherapy in patients with advanced cancer and a germline BRCA1/2 mutation. J Clin Oncol.

[24] Domchek SM, Aghajanian C, Shapira-Frommer R, Schmutzler RK, Audeh MW, Friedlander M, Balmaña J, Mitchell G, Fried G, Stemmer SM, Hubert A, Rosengarten O, Loman N (2016). Efficacy and safety of olaparib monotherapy in germline BRCA1/2 mutation carriers with advanced ovarian cancer and three or more lines of prior therapy. Gynecol Oncol.

[25] Rebbeck TR, Friebel T, Lynch HT, Neuhausen SL, van ’t Veer L, Garber JE, Evans GR, Narod SA, Isaacs C, Matloff E, Daly MB, Olopade OI, Weber BL (2004). Bilateral prophylactic mastectomy reduces breast cancer risk in BRCA1 and BRCA2 mutation carriers: the PROSE Study Group. J Clin Oncol.

[26] Domchek SM, Friebel TM, Singer CF, Evans DG, Lynch HT, Isaacs C, Garber JE, Neuhausen SL, Matloff E, Eeles R, Pichert G, t’veer L Van, Tung N (2010). Association of risk-reducing surgery in BRCA1 or BRCA2 mutation carriers with cancer risk and mortality. Jama.

[27] Kaas R, Verhoef S, Wesseling J, Rookus MA, Oldenburg HS, Peeters MJ, Rutgers EJ (2010). Prophylactic mastectomy in BRCA1 and BRCA2 mutation carriers: very low risk for subsequent breast cancer. Ann Surg.

[28] Kauff ND, Satagopan JM, Robson ME, Scheuer L, Hensley M, Hudis CA, Ellis NA, Boyd J, Borgen PI, Barakat RR, Norton L, Castiel M, Nafa K (2002). Risk-reducing salpingo-oophorectomy in women with a BRCA1 or BRCA2 mutation. N Engl J Med.

[29] Rebbeck TR, Lynch HT, Neuhausen SL, Narod SA, Veer LV, Garber JE, Evans G, Isaacs C, Daly MB, Matloff E, Olopade OI, Weber BL, Prevention (2002). Prophylactic oophorectomy in carriers of BRCA1 or BRCA2 mutations. N Engl J Med.

[30] King MC, Wieand S, Hale K, Lee M, Walsh T, Owens K, Tait J, Ford L, Dunn BK, Costantino J, Wickerham L, Wolmark N, Fisher B (2001). Tamoxifen and breast cancer incidence among women with inherited mutations in BRCA1 and BRCA2: National Surgical Adjuvant Breast and Bowel Project (NSABP-P1) Breast Cancer Prevention Trial. Jama.

[31] de Groot JS, Moelans CB, Elias SG, Jo Fackler M, van Domselaar R, Suijkerbuijk KPM, Witkamp AJ, Sukumar S, van Diest PJ, van der Wall E (2016). DNA promoter hypermethylation in nipple fluid: a potential tool for early breast cancer detection. Oncotarget.

[32] Warner E, Hill K, Causer P, Plewes D, Jong R, Yaffe M, Foulkes WD, Ghadirian P, Lynch H, Couch F, Wong J, Wright F, Sun P (2011). Prospective study of breast cancer incidence in women with a BRCA1 or BRCA2 mutation under surveillance with and without magnetic resonance imaging. J Clin Oncol.

[33] Passaperuma K, Warner E, Causer PA, Hill KA, Messner S, Wong JW, Jong RA, Wright FC, Yaffe MJ, Ramsay EA, Balasingham S, Verity L, Eisen A (2012). Long-term results of screening with magnetic resonance imaging in women with BRCA mutations. Br J Cancer.

[34] Kriege M, Brekelmans CT, Boetes C, Besnard PE, Zonderland HM, Obdeijn IM, Manoliu RA, Kok T, Peterse H, Tilanus-Linthorst MM, Muller SH, Meijer S, Oosterwijk JC (2004). Efficacy of MRI and mammography for breast-cancer screening in women with a familial or genetic predisposition. N Engl J Med.

[35] Warner E, Plewes DB, Hill KA, Causer PA, Zubovits JT, Jong RA, Cutrara MR, DeBoer G, Yaffe MJ, Messner SJ, Meschino WS, Piron CA, Narod SA (2004). Surveillance of BRCA1 and BRCA2 mutation carriers with magnetic resonance imaging, ultrasound, mammography, and clinical breast examination. Jama.

[36] Leach MO, Boggis CR, Dixon AK, Easton DF, Eeles RA, Evans DG, Gilbert FJ, Griebsch I, Hoff RJ, Kessar P, Lakhani SR, Moss SM, Nerurkar A (2005). Screening with magnetic resonance imaging and mammography of a UK population at high familial risk of breast cancer: a prospective multicentre cohort study (MARIBS). Lancet.

[37] Kuhl CK, Schrading S, Leutner CC, Morakkabati-Spitz N, Wardelmann E, Fimmers R, Kuhn W, Schild HH (2005). Mammography, breast ultrasound, and magnetic resonance imaging for surveillance of women at high familial risk for breast cancer. J Clin Oncol.

[38] Okugawa H, Yamamoto D, Uemura Y, Sakaida N, Tanano A, Tanaka K, Kamiyama Y (2005). Effect of perductal paclitaxel exposure on the development of MNU-induced mammary carcinoma in female S-D rats. Breast Cancer Res Treat.

[39] Murata S, Kominsky SL, Vali M, Zhang Z, Garrett-Mayer E, Korz D, Huso D, Baker SD, Barber J, Jaffee E, Reilly RT, Sukumar S (2006). Ductal access for prevention and therapy of mammary tumors. Cancer Res.

[40] Stearns V, Mori T, Jacobs LK, Khouri NF, Gabrielson E, Yoshida T, Kominsky SL, Huso DL, Jeter S, Powers P, Tarpinian K, Brown RJ, Lange JR (2011). Preclinical and clinical evaluation of intraductally administered agents in early breast cancer. Sci Transl Med.

[41] Love SM, Zhang W, Gordon EJ, Rao J, Yang H, Li J, Zhang B, Wang X, Chen G, Zhang B (2013). A feasibility study of the intraductal administration of chemotherapy. Cancer Prev Res (Phila).

[42] Mahoney ME, Gordon EJ, Rao JY, Jin Y, Hylton N, Love SM (2013). Intraductal therapy of ductal carcinoma in situ: a presurgery study. Clin Breast Cancer.

[43] Derksen PW, Braumuller TM, van der Burg E, Hornsveld M, Mesman E, Wesseling J, Krimpenfort P, Jonkers J (2011). Mammary-specific inactivation of E-cadherin and p53 impairs functional gland development and leads to pleomorphic invasive lobular carcinoma in mice. Dis Model Mech.

[44] Deurloo MJ, Kop W, van Tellingen O, Bartelink H, Begg AC (1991). Intratumoural administration of cisplatin in slow-release devices: II. Pharmacokinetics and intratumoural distribution. Cancer Chemother Pharmacol.

[45] Schedin P, Mitrenga T, Kaeck M (2000). Estrous cycle regulation of mammary epithelial cell proliferation, differentiation, and death in the Sprague-Dawley rat: a model for investigating the role of estrous cycling in mammary carcinogenesis. J Mammary Gland Biol Neoplasia.

[46] Hvid H, Thorup I, Sjogren I, Oleksiewicz MB, Jensen HE (2012). Mammary gland proliferation in female rats: effects of the estrous cycle, pseudo-pregnancy and age. Exp Toxicol Pathol.

[47] Blagosklonny MV (2005). Carcinogenesis, cancer therapy and chemoprevention. Cell Death Differ.

[48] Chun YS, Yoshida T, Mori T, Huso DL, Zhang Z, Stearns V, Perkins B, Jones RJ, Sukumar S (2012). Intraductally administered pegylated liposomal doxorubicin reduces mammary stem cell function in the mammary gland but in the long term, induces malignant tumors. Breast Cancer Res Treat.

[49] Gietema JA, Meinardi MT, Messerschmidt J, Gelevert T, Alt F, Uges DR, Sleijfer DT (2000). Circulating plasma platinum more than 10 years after cisplatin treatment for testicular cancer. Lancet.

[50] Brouwers EE, Huitema AD, Beijnen JH, Schellens JH (2008). Long-term platinum retention after treatment with cisplatin and oxaliplatin. BMC Clin Pharmacol.

[51] Bergfeldt K, Einhorn S, Rosendahl I, Hall P (1995). Increased risk of second primary malignancies in patients with gynecological cancer. A Swedish record-linkage study. Acta Oncol.

[52] Travis LB, Curtis RE, Boice JD, Platz CE, Hankey BF, Fraumeni JF (1996). Second malignant neoplasms among long-term survivors of ovarian cancer. Cancer Res.

[53] van den Belt-Dusebout AW, de Wit R, Gietema JA, Horenblas S, Louwman MW, Ribot JG, Hoekstra HJ, Ouwens GM, Aleman BM, van Leeuwen FE (2007). Treatment-specific risks of second malignancies and cardiovascular disease in 5-year survivors of testicular cancer. J Clin Oncol.

[54] Fung C, Fossa SD, Milano MT, Oldenburg J, Travis LB (2013). Solid tumors after chemotherapy or surgery for testicular nonseminoma: a population-based study. J Clin Oncol.

[55] Hamelers IH, Staffhorst RW, Voortman J, de Kruijff B, Reedijk J (2009). van Bergen en Henegouwen PM, de Kroon AI. High cytotoxicity of cisplatin nanocapsules in ovarian carcinoma cells depends on uptake by caveolae-mediated endocytosis. Clin Cancer Res.

[56] Casagrande N, De Paoli M, Celegato M, Borghese C, Mongiat M, Colombatti A, Aldinucci D (2013). Preclinical evaluation of a new liposomal formulation of cisplatin, lipoplatin, to treat cisplatin-resistant cervical cancer. Gynecol Oncol.

[57] Dickey DT, Wu YJ, Muldoon LL, Neuwelt EA (2005). Protection against cisplatin-induced toxicities by N-acetylcysteine and sodium thiosulfate as assessed at the molecular, cellular, and in vivo levels. J Pharmacol Exp Ther.

[58] Liu X, Holstege H, van der Gulden H, Treur-Mulder M, Zevenhoven J, Velds A, Kerkhoven RM, van Vliet MH, Wessels LF, Peterse JL, Berns A, Jonkers J (2007). Somatic loss of BRCA1 and p53 in mice induces mammary tumors with features of human BRCA1-mutated basal-like breast cancer. Proc Natl Acad Sci U S A.

[59] Sleeman KE, Kendrick H, Robertson D, Isacke CM, Ashworth A, Smalley MJ (2007). Dissociation of estrogen receptor expression and in vivo stem cell activity in the mammary gland. J Cell Biol.

[60] Brouwers EE, Tibben MM, Rosing H, Hillebrand MJ, Joerger M, Schellens JH, Beijnen JH (2006). Sensitive inductively coupled plasma mass spectrometry assay for the determination of platinum originating from cisplatin, carboplatin, and oxaliplatin in human plasma ultrafiltrate. J Mass Spectrom.

