# Supplementary material for: Intraductal cisplatin treatment in a BRCA-associated breast cancer mouse model attenuates tumor development but leads to systemic tumors in aged female mice
Source: Oncotarget. 2017 Jun 15;8(37):60750–63. doi: 10.18632/oncotarget.18490 (PMC5617383; doi:10.18632/oncotarget.18490)
Supplement: Supplementary file 1 [file oncotarget-08-60750-s001.pdf]

Intraductal cisplatin treatment in a *BRCA*-associated breast cancer mouse model attenuates tumor development but leads to systemic tumors in aged female mice

Supplementary Material

A

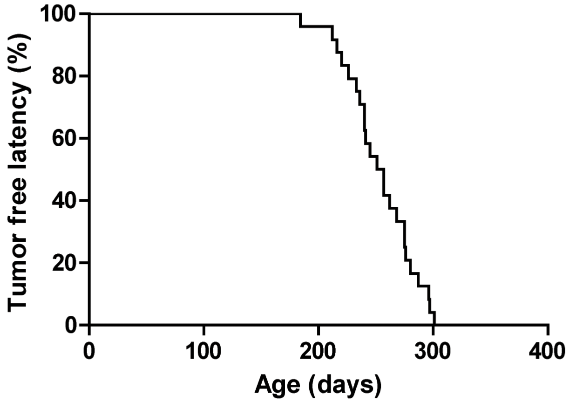

B

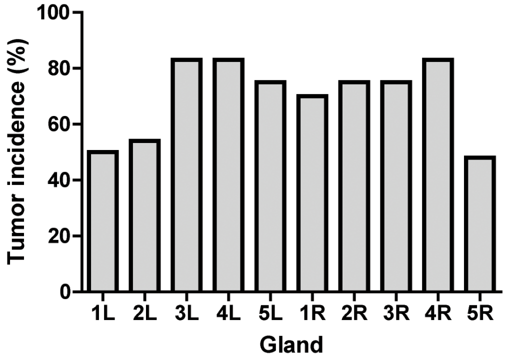

Supplementary Figure 1: Tumor latency and incidence in control WAPCre;Brca1F/F;Trp53F/F.
(A) Tumor latency in control mice (n=24) in days after birth.
(B) Breast tumor incidence in control mice depicted for the separate mammary glands.

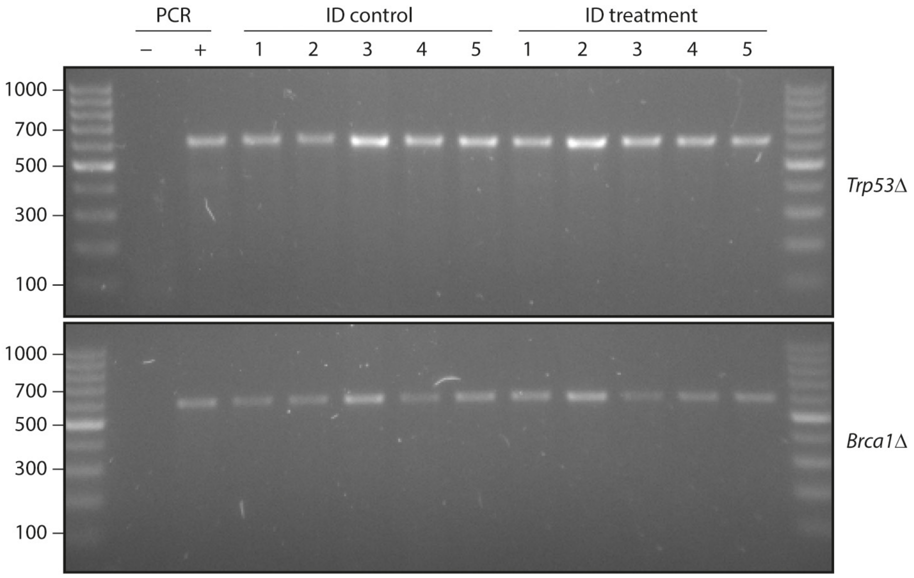

Supplementary Figure 2: PCR results for floxed regions of Brca1 and Trp53. PCR results from from tumor DNA for ΔTrp53 (upper row) and ΔBrca1 (lower row) of control mice or mice treated with ID cisplatin. In the first two lanes negative and positive control are loaded, respectively.

For Supplementray Tables see in Supplementary Files
