# Supplementary material for: Intraductal cisplatin treatment in a BRCA-associated breast cancer mouse model attenuates tumor development but leads to systemic tumors in aged female mice
Source: Oncotarget. 2017 Jun 15;8(37):60750–63. doi: 10.18632/oncotarget.18490 (PMC5617383; doi:10.18632/oncotarget.18490)
Supplement: Supplementary file 2 [file oncotarget-08-60750-s002.docx]

**Supplementary Table 1:** Additional pathology *WAPcre;Brca1F/F;Trp53F/F* mice.

| **Histology** | **Control**  **N=24** | **Cisplatin**  **N=20** | **Olaparib**  **N=29** | **Combination**  **N=27** |
| --- | --- | --- | --- | --- |
| Lymph node metastases | 0 (0.0%) | 0 (0.0%) | 0 (0.0%) | 2 (7.4%) |
| Lung metastases | 5 (20.8%) | 7 (35.0%) | 4 (13.7%) | 1 (3.7%) |
| Liver metastases | 0 (0.0%) | 0 (0.0%) | 0 (0.0%) | 1 (3.7%) |
| Leukemia | 7 (29.2%) | 5 (25.0%) | 9 (31.0%) | 5 (18.5%) |
| Adenocarcinoma lung | 0 (0.0%) | 0 (0.0%) | 0 (0.0%) | 1 (3.7%) |
| Sarcoma unknown origin with multiple metastases lung, liver and spleen | 0 (0.0%) | 0 (0.0%) | 0 (0.0%) | 1 (3.7%) |

All found additional pathology did not significantly differ between treatment groups.**Supplementary Table 2:** Additional pathology aged wild type mice.

| **Organ** | **Histology** | **Control^1^** | **Cisplatin^1^** | **p-value** |
| --- | --- | --- | --- | --- |
| Mammary gland | Secretion | 21/64 | 43/84 | **0.025** |
|  | Ductal distension | 32/64 | 43/84 | 0.886 |
|  | Vessel distension | 1/64 | 1/84 | 0.846 |
|  | Squamous metaplasia locally treated glands | 15/64 | 17/84 | 0.639 |
|  | Squamous metaplasia untreated glands | 9/64 | 14/84 | 0.865 |
|  | Benign tumor | 3/64 | 8/84 | 0.352 |
|  | Adenosis | 2/64 | 6/84 | 0.467 |
|  | Periductitis | 8/64 | 7/84 | 0.405 |
|  | Lobulitis | 1/64 | 6/84 | 0.467 |
|  | Ductal hyperplasia | 0/64 | 5/84 | 0.070 |
|  | Fat cell necrosis | 2/64 | 0/84 | 0.185 |
|  | Adnex tumor benign | 20/64 | 9/84 | **0.002** |
|  | Haemangioma | 0/66 | 1/85 | 1.000 |
|  | Pseudo lymph node | 1/66 | 0/85 | 0.437 |
|  | Ectopic parotis | 0/64 | 1/84 | 1.000 |
| Pituitary gland | Adenoma/hyperplasia | 7/57 | 14/73 | 0.289 |
| Salivary gland | Benign oncocytoma | 0/67 | 1/86 | 1.000 |
|  | Parotitis | 0/67 | 1/86 | 1.000 |
| Sinus | Papilloma | 10/67 | 15/86 | 0.676 |
| Skin | Ulcerative dermatitis | 0/67 | 1/86 | 1.000 |
|  | Hyperkeratotic papilloma | 0/67 | 1/86 | 1.000 |
|  | Sebaceous gland adenoma (outside MG) | 0/67 | 1/86 | 1.000 |
| Heart | Thrombus | 0/67 | 1/85 | 1.000 |
|  | Infiltration neutrophils | 1/67 | 2/85 | 1.000 |
|  | Fibrosis | 1/67 | 0/85 | 0.441 |
|  | Fat cell infiltration myocardium | 1/67 | 0/85 | 0.441 |
| Lungs | Infiltration neutrophils | 0/64 | 3/85 | 0.260 |
|  | Hyperplasia type II pneumocytes | 0/64 | 4/84 | 0.134 |
|  | Bacterial infiltration | 1/64 | 0/85 | 0.430 |
| Mediastinal fat | Infiltration neutrophils | 1/64 | 0/85 | 0.430 |
| Liver | Steatosis | 39/63 | 38/83 | 0.066 |
|  | Altered foci | 2/63 | 7/83 | 0.300 |
|  | Hyperplasia/dysplasia | 1/66 | 0/86 | 0.434 |
|  | Adenoma | 0/63 | 2/83 | 0.506 |
|  | Necrosis | 2/63 | 2/83 | 1.000 |
|  | Biliary cyst formation | 1/63 | 1/83 | 1.000 |
| Spleen | Hemorrhage | 1/62 | 0/83 | 0.428 |
| Kidneys | Lympho-plasmacytic aggregates | 43/62 | 73/85 | **0.015** |
|  | Periglomerular cyst formation | 8/63 | 27/85 | **0.007** |
|  | Cyst formation other (tubular) | 3/63 | 3/85 | 0.700 |
|  | Pyelum distension | 2/63 | 1/85 | 0.575 |
|  | Infarction | 2/63 | 1/85 | 0.575 |
|  | Mineralization renal papilla | 0/63 | 3/85 | 0.262 |
| Intestines | Inflammation | 1/62 | 0/81 | 0.434 |
|  | Polyps/adenomas | 2/62 | 1/81 | 0.579 |
| Mesenterium | Steatitis/necrosis | 4/62 | 1/81 | 0.166 |
| Uterus/ovaries | Hydrouterus | 18/64 | 14/85 | 0.086 |
|  | Hematocolpos/thrombus | 8/64 | 4/85 | 0.716 |
|  | Benign tumor | 6/64^2^ | 9/85^3^ | 0.808 |
|  | Necrosis | 1/64 | 0/85 | 0.430 |
|  | Cyst formation | 2/64 | 6/85 | 0.467 |
|  | Polyp formation | 0/64 | 2/85 | 0.507 |
|  | Endometritis | 2/64 | 1/85 | 0.577 |
|  | Salpingitis | 1/64 | 0/85 | 0.430 |
|  | Cervicitis | 0/64 | 1/85 | 1.000 |
| Blood vessels | Multisystemic vasculitis (PAN) | 3/66 | 3/86 | 1.000 |
| Lymph nodes | Sinus histiocytosis | 0/67 | 2/86 | 0.504 |
| Hematology | EMH | 10/65 | 25/85 | **0.044** |
|  | Myeloid hyperplasia | 10/63 | 7/82 | 0.173 |
|  | Lymphoid hyperplasia | 5/63 | 12/82 | 0.214 |
|  | Erythroid hyperplasia | 8/65 | 9/86 | 0.723 |
| Bone | New bone formation bone marrow femur | 1/65 | 9/85 | **0.043** |

^1^ Incidence is described per total number of analyzed slides.

^2^ This group consists of: leimyoma uterus, Leydig cell hyperplasia uterus, spindle cell lipoma uterus, hemangioma (1 uterus, 1 tuba), corpora albicantia.

^3^ This group consists of: leiomyoma uterus (2), myoma uterus, adenomyoma uterus, Leydig cell hyperplasia/tumor uterus (2), stromal nodus uterus, paracervical myoma, benign tumor ovary without clear diagnosis.
